# Exosomes Derived from BMMSCs Mitigate the Hepatic Fibrosis via Anti-Pyroptosis Pathway in a Cirrhosis Model

**DOI:** 10.3390/cells11244004

**Published:** 2022-12-10

**Authors:** Yichi Zhang, Hanjing Zhangdi, Xinsheng Nie, Lijuan Wang, Zhuzhi Wan, Hao Jin, Ronghui Pu, Meihui Liang, Yuan Chang, Yang Gao, Hailong Zhang, Shizhu Jin

**Affiliations:** 1 Department of Gastroenterology and Hepatology, The Second Affiliated Hospital, Harbin Medical University, Harbin 150086, China; 2 The Xinjiang Production and Construction Corps Tenth Division Beitun Hospital, Beitun 806099, China

**Keywords:** Exo-rBMMSC, BMMSCs, liver cirrhosis, pyroptosis

## Abstract

Researchers increasingly report the therapeutic effect of exosomes derived from rat bone marrow mesenchymal stem cells (Exos-rBMMSC) on liver disease, while the optimal dose of Exos-rBMMSC in liver cirrhotic treatment has not been reported. In this study, we aimed to explore the efficacy and dose of Exos-rBMMSC in a hepatic cirrhosis rat model. The therapeutic effects of a low dose, medium dose and high dose of Exos-rBMMSC were assessed by liver function tests and histopathology. After four-weeks of Exos-rBMMSC therapy, pyroptosis-related expression levels in the medium dose and the high dose Exos-rBMMSC groups were significantly decreased compared to those in the liver cirrhosis group (*p* < 0.05). The hepatic function assay and histopathology results showed significant improvement in the medium dose and the high dose Exos-rBMMSCs groups. The localization of PKH67-labeled Exos-rBMMSC was verified microscopically, and these particles were coexpressed with the PCNA, NLRP3, GSDMD and Caspase-1. Our results demonstrated that Exos-rBMMSC accelerated hepatocyte proliferation and relieved liver fibrosis by restraining hepatocyte pyroptosis. More importantly, we confirmed that the high dose of Exos-rBMMSC may be the optimal dose for liver cirrhosis, which is conducive to the application of Exos-rBMMSC as a promising cell-free strategy.

## 1. Introduction

Liver cirrhosis is the terminal phase of all types of chronic hepatic diseases. Currently, without an effective therapy for liver cirrhosis, patients are more likely to experience hospital readmissions, hepatocellular carcinoma and death, which results in a substantial economic burden. The annual death rate from liver cirrhosis worldwide is as high as 1.32 million [1]. Therefore, developing an effective therapy is an urgent problem to be solved.

In recent years, accumulating evidence from preclinical trials has demonstrated that rat bone marrow mesenchymal stem cell (rBMMSC) transplantation can alleviate collagen accumulation and improve liver function [2,3]. However, the therapeutic effect is limited by low differentiation efficiency, and most stem cells are obstructed in the pulmonary vasculature [4]. Moreover, the clinical application of stem cell transplantation is hampered by complications such as tumorigenicity and immunologic rejection [5]. Hence, exosomes (Exos), which possess similar biological topology as stem cells, are important paracrine mediators and have become a promising and advanced cell-free remedy. Exosomes are vesicles that are 30~150 nm in diameter, are secreted by all kinds of cells, and transfer functional miRNAs, mRNAs, proteins and cytokines, and these vesicles have inspired substantial enthusiasm for research into their potential roles in mediating intercellular metastasis [6,7]. However, few studies have focused on the applicable doses of exosomes, which is a gap that hampers the development of practical clinical applications.

In this study, we aimed to explore the optimal dose of exosomes derived from rBMMSCs (Exo-rBMMSC) for treating liver cirrhosis. The therapeutic effects of the low dose, the medium dose and the high dose Exo-rBMMSC were evaluated by examining pyroptosis inhibition, pathological histology, liver function and molecular biology in vivo and in vitro. In addition, the distribution of Exo-rBMMSC was examined by near-infrared fluorescence (NIRF) imaging. Overall, our results suggested that Exo-rBMMSC alleviated liver cirrhosis and enhanced hepatic function.

## 2. Materials and Methods

### 2.1. Establishment of the Rat Liver Cirrhosis Model

Sprague-Dawley rats (130~150 g, male) were obtained from the animal facility of the Second Affiliated Hospital of Harbin Medical University. A liver cirrhosis model was established as we described previously [3]. Briefly, 50% CCl_4_ 0.1 mL/100 g (dissolved in oil) was intraperitoneally injected twice each week for 8 weeks. After 8 weeks of stimulation, the animals were examined by histopathology. In addition, all experiments and methods were approved by the Experimental Centre of the Second Affiliated Hospital of Harbin Medical University (Ethics Project Number: KY2020-144).

### 2.2. Cell Culture

The purification and identification of rBMMSCs were described in our previous study [3]. rBMMSCs were cultured in DMEM/F12 medium (Sigma, St. Louis, MO, USA), 10% FBS (Corning, NYC, New York, NY, USA), and 1% penicillin–streptomycin (pH = 7.4, Sigma, St. Louis, MO, USA). Exo-rBMMSCs were collected from third-passage rBMMSCs to fifth-passage rBMMSCs. BRL rat hepatocytes were purchased from BEINA Biology Company and cultured in MEM (Sigma, St. Louis, MO, USA) and 10% FBS.

To further verify the activation of the pyroptosis signaling pathway in the liver cirrhosis model, disulfiram dissolved in DMSO (0.3 μM, MedChemExpress, Monmouth Junction, NJ, USA) was added into hepatocytes for 3 h prior to the induction of pyroptosis [8]. After the incubation with CCl_4_ (10 mM, dissolved in DMSO) for 24 h, the pyroptosis related proteins expression levels of hepatocytes were assayed, and the IL-1β and IL-18 expression level of hepatocytes supernatant were examined by ELISA. To exclude the cytotoxicity of DMSO, we also examined the expression level of pyroptosis related proteins in hepatocytes and of the IL-1β and IL-18 in hepatocytes supernatant.

### 2.3. Cell Viability Analysis

The viability of untreated hepatocytes, hepatocytes treated with CCl_4_ (10 mM) and PBS, and hepatocytes treated with CCl_4_ (10 mM) and 100 μg Exo-rBMMSC was assessed by CCK-8 (APExBIO, Houston, TX, USA) and 5-ethynyl-2′-deoxyuridine (EdU) imaging kits (UE, Suzhou, China). Briefly, 5 × 10^3^ hepatocytes were seeded in 96-well plates for 24 h (n = 15). Then, CCl_4_ (10 mM) was added to 10 plates, and the same volume of PBS was added to 5 plates. After 12 h of incubation, the medium was replaced with MEM + 100 μg Exos or MEM + PBS. Then, after 48 h incubation, cell viability was measured according to the manufacturer’s protocol.

### 2.4. Experimental Groups Design

The liver cirrhotic rats were randomly divided into 5 groups (n = 5 for each group): (1) Control; (2) Liver cirrhosis; (3) Low dose Exos (100 μg exosomes harvested from 0.5 × 10^8^ rBMMSC); (4) Medium dose Exos (200 μg exosomes harvested from 1 × 10^8^ rBMMSC); (5) High dose Exos (400 μg exosomes harvested from 2 × 10^8^ rBMMSC). 100, 200, 400 μg total protein of exosomes dissolved in 1 mL PBS per rat were injected by the tail vein twice a week for 4 weeks, while the rats with liver cirrhosis alone were injected with an equal volume of PBS.

### 2.5. Purification and Characterization of Exo-rBMMSC

rBMMSC culture supernatant was collected after the cells were cultured in exosomes-free medium (DMEM/F12, 10% exosome-free FBS, 1% penicillin-streptomycin) for 24 h. Then, exosomes were purified according to the protocol in the Journal of Extracellular Vesicles [9,10]. Briefly, the supernatant was concentrated by differential ultracentrifugation, followed by centrifugation at 300× *g* for 10 min, 2000× *g* for 10 min, and 10,000× *g* for 30 min. Finally, the supernatant was ultracentrifuged at 100,000 × *g* for 70 min twice. The precipitate was resuspended in 200 μL of phosphate-buffered saline (PBS). The protein concentration of Exo-rBMMSCs was measured by a BCA assay [11]. To characterize the purified vesicles as exosomes, the morphology was examined by transmission electronic microscopy (TEM), and the diameters were measured and analyzed by a Zeta View Particle Metrix (Zeta View PMX 110). The exosomal protein markers CD9, TSG101, and TSG70 (1:1000, Abcam, Massachusetts, CB, USA) and the negative marker calnexin (1:1000, Abcam, St. Massachusetts, CB, USA) were examined by Western blotting [12,13].

### 2.6. PKH67 Staining

According to the protocol and the published study, to prepare the 2× dye solution, 2 µL of PKH67 ethanolic dye was added to 1 mL of Diluent C ([14], Sigma-Aldrich, St. Louis, MO, USA). Then, 1 mL of the exosomes solution was rapidly added into the 2× dye solution and mixed slowly for 4 min. To suspend redundant staining, 2 mL of serum-free FBS was added. PBS was added to adjust the volume to 14 mL, and the mixture was centrifuged at 100,000× *g* at 4 °C for 90 min. The supernatant was removed, and PKH67-labeled Exo-rBMMSCs were resuspended in 200 μL of PBS. Similarly, rBMMSCs were stained with PKH67 according to the exosome procedure.

### 2.7. Coculture of Hepatocytes and rBMMSC

The transwell coculture system (Corning Costar, NYC, New York, NY, USA) consisted of 1 × 10^6^ hepatocytes and 1 × 10^6^ rBMMSCs. Hepatocytes were seeded in 6-well plates, and PKH67-labeled rBMMSCs were cultured in Transwell inserts. After 24 h of coculture, the hepatocytes were observed under a fluorescence microscope (Zeiss-DMI8, Oberkochen, St. Baden-Württemberg, German).

### 2.8. Exosomes Uptake Assessment

PKH67-labelled Exo-rBMMSC (100 μg) were added to hepatocytes and cultured in 6-well plates with FBS-free medium. After 24 h of incubation, the hepatocytes were assessed by fluorescence microscopy.

### 2.9. Biodistribution of Exo-rBMMSC

To detect the distribution of the Exo-rBMMSC in vivo, Exo-rBMMSC were stained with 1,1′-dioctadecyl-3,3,3′,3′-tetramethylindotricarbocyanine iodide (DiR, D12731, Invitrogen, Life Technologies, Carlsbad, CA, USA), which was obtained commercially from Sigma. DiR-stained Exo-rBMMSC (100 μg) were injected into CCl_4_-induced rats. At 0, 3 and 24 h after tail vein injection, ex vivo fluorescent images of different organs, including the heart, lung, liver, spleen and kidney, were taken by an in vivo imaging system (Night OWL II LB983, Berthold Technologies, Wildbad, Germany).

### 2.10. Immunofluorescence and Immunohistochemical Assays

Immunofluorescence and immunohistochemical examinations were conducted as described previously [3]. Briefly, frozen liver sections (4 μm) were used for immunofluorescence assays, and paraffin liver sections (4 μm) were used for immunohistochemical assays. Primary antibodies against NLRP3 (1:50, NOVUS, Centennial, CO, USA), GSDMD (1:1000, Abcam, CB, Waltham, MA, USA), Caspase-1 (1:200, Sigma, St. Louis, MO, USA), and PCNA (1:200, Affinity Biosciences, Cincinnati, OH, USA) were added and incubated overnight at 4 °C. Then, immunofluorescence images were taken by fluorescence microscopy (Zeiss-DMI8), and immunohistochemical images were obtained by an Olympus (BX41) microscope and semiquantitatively measured by Fiji software.

### 2.11. Western Blotting

The Western blot procedure was described in our previous study [3]. Briefly, proteins were extracted from frozen liver tissue. After electrophoresis, the proteins were transferred to nitrocellulose membranes, which were incubated with β-actin (1:1000, Abcam, Massachusetts, CB, USA), NLRP3 (1:5000, NOVUS), GSDMD (1:1000, Abcam, CB, Waltham, MA, USA), cleaved caspase-1 (1:1000, Cell Signaling Technology, Boston, MA, USA), and IL-1β (1:1000, Abclonal, Wuhan, China) antibodies overnight at 4 °C. Then, the nitrocellulose membranes were incubated with HRP-conjugated secondary antibodies (1:5000, Boster, Wuhan, China). Finally, images were taken using the ImageQuant LAS 4000 mini machine (GE).

### 2.12. ELISA

Rat tissue and serum samples were obtained after euthanasia. Fresh frozen hepatic tissues were homogenized with PBS at 4 °C according to the manufacturer’s protocol. After centrifugation at 120,000× *g* at 4 °C for 20 min, the supernatant was collected. Liver function was evaluated by measuring the levels of ALT, AST and ALB with ELISA kits (Nanjing Jiancheng Bioengineering Institute, Nanjing, China). The IL-1β and IL-18 levels in serum and cell supernatants were examined by ELISA kits (Boster, China) according to the protocol.

### 2.13. Histopathological Assays

The liver tissues were fixed with 4% paraformaldehyde and embedded in paraffin. Liver sections (4 μm) were stained with hematoxylin and eosin (HE) to observe pathological morphology and with Masson’s trichrome to examine collagen deposition. Semi-quantitative analysis was performed by Fiji software.

### 2.14. Statistical Analyses

GraphPad Prism 9 software (Version 9.0.1, Inc.) was used to analyze the data by one-way analysis of variance (ANOVA). All data are displayed as the mean ± standard deviation. Statistical significance was defined as *p* < 0.05 (*/^#^ *p* < 0.05; **/^##^ *p* < 0.01; ***/^###^ *p* < 0.001; ****/^####^ *p* < 0.0001; ns, not significant). All figures were prepared by GraphPad Prism 9 and Photoshop 7.1.

## 3. Results

### 3.1. Liver Cirrhosis Model Establishment and rBMMSC Purification

Liver cirrhosis models were induced by intraperitoneal injections of 50% CCl_4_ diluted in olive oil (5.0 mL/kg) and assessed by liver function tests, histological analysis and serological hydroxyproline determination, and the results indicated that the liver cirrhosis models were successfully established in our previous study [3]. Additionally, rBMMSCs were extracted successfully and identified by morphology, osteogenic and adipogenic differentiation and flow cytometry in our previous study [3].

Pyroptosis-related proteins are highly expressed in liver cirrhosis and can be alleviated by rBMMSCs.

**Figure 1:**
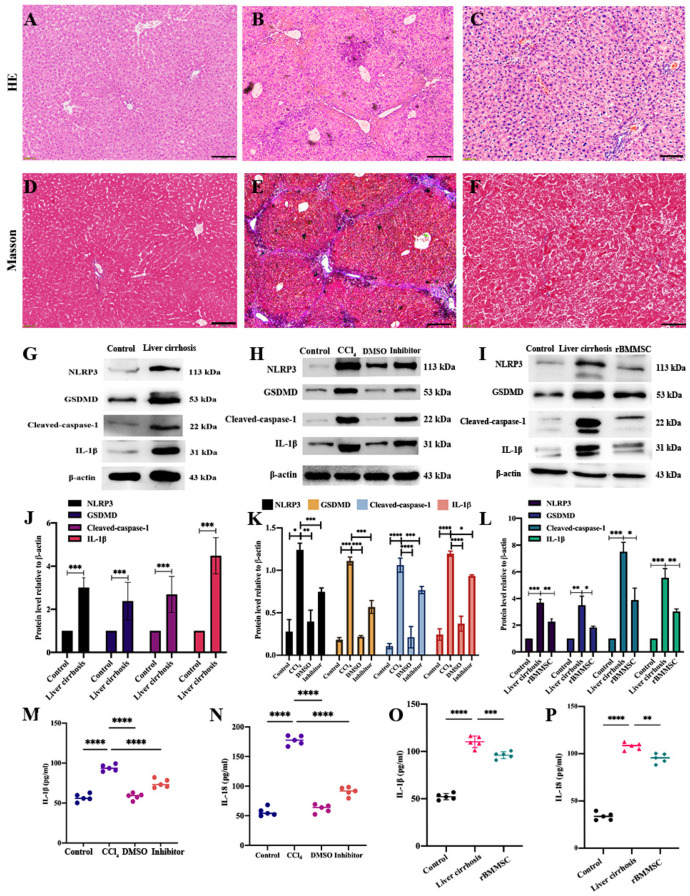
The alleviation of pyroptosis by rBMMSCs in the context of liver cirrhosis. Pathological HE- and Masson-stained sections of control and cirrhotic liver tissues are shown (**A**–**F**). Pathological sections showed significant alleviation in the rBMMSC transplantation group (**B**,**C**). Scale bar = 100 μm applied to (**A**–**F**). Pyroptosis-related proteins (NLRP3, GSDMD, cleaved caspase-1 and IL-1β) were markedly highly expressed in the liver cirrhosis model ((**G**,**J**), *** *p* < 0.001 vs. the control). The disulfram inhibited the expression of pyroptosis-related proteins, as shown in (**H**). The expression levels of pyroptosis-related proteins in the inhibitor group were significantly decreased compared to those in the CCl_4_ group ((**K**), * *p* < 0.05). The IL-1β and IL-18 expression in the inhibitor group were significantly down-regulated compared to those in the CCl_4_ group ((**M**,**N**), **** *p* < 0.0001). Pyroptosis-related proteins in the BMMSC group were significantly down-regulated compared to those in the liver cirrhosis group ((**I**,**L**), * *p* < 0.05). IL-1β and IL-18 expression in the BMMSC group were dramatically decreased ((**O**,**P**), ** *p* < 0.01 vs. the liver cirrhosis group). The data are displayed as the means ± SD; n = 5 for each group.

HE-stained cirrhotic liver sections showed cellular necrosis, inflammatory cell infiltration and pseudolobuli formation compared to those in the control group (Figure 1A,B). Masson staining of sections showed that compared to those in the control group, hepatic collagen production and collagen deposition were obviously present (Figure 1D,E). After 4 weeks of intraperitoneal injections of 50% CCl_4_, pyroptosis-related proteins were significantly highly expressed in the liver cirrhosis group compared to the control group (Figure 1G,J, *** *p* < 0.001). To further explore the effect of pyroptosis on liver cirrhosis, the pyroptosis pathway specific inhibitor disulfiram was co-cultured with hepatocytes for 3 h. The pyroptosis-related proteins (NLRP3, GSDMD, cleaved-caspase-1 and IL-1β) of hepatocytes in the inhibitor group were significantly decreased compared to the CCl_4_ group (* *p* < 0.05, Figure 1H,K). The IL-1β and IL-18 expression levels of hepatocytes supernatant were assayed by ELISA. Compared to the CCl_4_ group, the expression levels of IL-1β and IL-18 in inhibitor group were significantly decreased (**** *p* < 0.0001, Figure 1M,N). In addition, 4 weeks of rBMMSC transplantation via caudal vein injection markedly restrained the expression of pyroptosis-related proteins (NLRP3, GSDMD, cleaved caspase-1 and IL-1β, Figure 1I,L, * *p* < 0.05). IL-1β and IL-18, which are pyroptosis-related inflammatory factors, were decreased after 4 weeks of rBMMSC transplantation (Figure 1N,O, ** *p* < 0.01).

### 3.2. Paracrine Effect of rBMMSCs on Inhibiting Hepatocyte Pyroptosis

**Figure 2:**
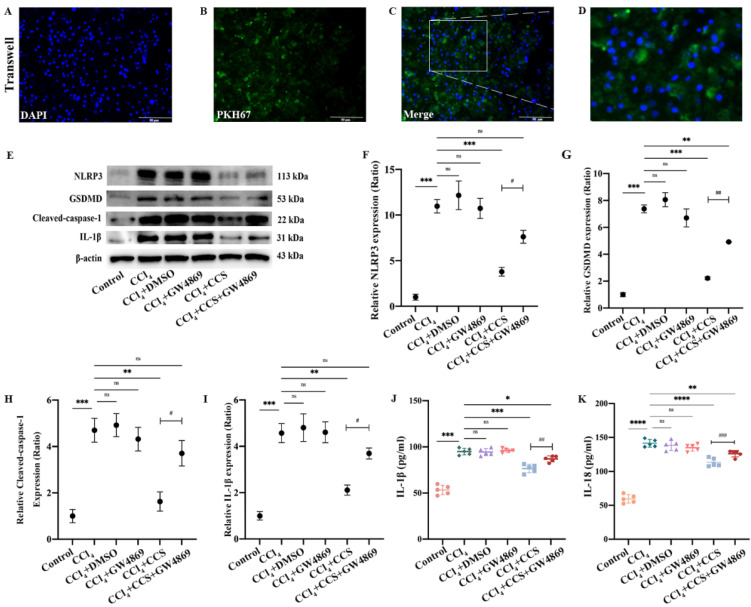
Paracrine effect by which rBMMSCs inhibit hepatocyte pyroptosis. PKH67 dye was transferred to hepatocytes by rBMMSCs via the paracrine pathway ((**A**–**D**), Scale bar = 50 μm). Pyroptosis-related proteins in the CCl_4_ + CCS group were significantly decreased compared to those in the CCl_4_ group ((**E**–**I**), ** *p* < 0.01). Pyroptosis-related proteins in the CCl_4_ + CCS+GW4869 group were significantly increased comparing with those in the CCl_4_ + CCS group ((**E**–**I**), # *p* < 0.05). The expression levels of pyroptosis-related proteins in the CCl_4_ + DMSO and CCl_4_ + GW4869 groups was not significantly different compared to that in the CCl_4_ group (**E**–**I**). Cell viability in the CCl_4_ + CCS group was significantly higher than that in the other groups ((**J**), ## *p* < 0.01). The expression of IL-1β in the CCl_4_ + CCS group was dramatically lower than those in the CCl_4_ group ((**J**), *** *p* < 0.001). Similar results had been showed in (**K**), the expression of IL-18 in the CCl_4_ + CCS group were significantly decreased compared to those in the CCl_4_ group (**** *p* < 0.0001). The data are displayed as the means ± SD; n = 5 for each group.

After hepatocytes and rBMMSCs were cocultured for 24 h, PKH67 dye was observed in hepatocytes, which indicated that rBMMSCs affected hepatocytes in a paracrine manner (Figure 2A–D). GW4869 (dissolved in DMSO, 10 μM) is an inhibitor of membrane neutral sphingomyelin, which participates in exosomes generation and release [15]. To assess the effect of Exo-rBMMSC on hepatocytes, GW4869 was added into the rBMMSCs culture exosome-free medium. After 24 h culture, the cell culture supernatant (CCS) of rBMMSC was collected and then added into the hepatocytes treated with 10 mM CCl_4_ (CCl_4_ + CCS-GW4869 group). At the same time, the CCS of rBMMSCs culture exosome-free medium was collected and added into hepatocytes treated with 10 mM CCl_4_ (CCl_4_ + CCS group). To exclude the effect of DMSO on hepatocytes, the CCl_4_ + DMSO group was established, which referred to the hepatocytes treated with CCl_4_ (10 mM) and 1% DMSO. Similarly, for excluding the effect of GW4869, the CCl_4_ + GW4869 group was established, which referred to hepatocytes were treated with CCl_4_ and GW4869. Generally, the following groups were established: control group (untreated hepatocytes), CCl_4_ group (hepatocytes were treated with 10 mM CCl_4_ (dissolved in DMSO)), CCl_4_ + DMSO group, CCl_4_ + GW4869 group, CCl_4_ + CCS group and CCl_4_ + CCS-GW4869 group. After 6 h incubation of hepatocytes cultured with different culture conditions, the pyroptosis related proteins (NLRP3, GSDMD, cleaved caspase-1 and IL-1β) were assayed. Pyroptosis-related protein expression levels in the CCl_4_ + CCS group were significantly decreased (Figure 2E–I, ** *p* < 0.01 vs. the CCl_4_ group). Importantly, the expression of NLRP3, GSDMD, cleaved caspase-1 and IL-1β in the CCl_4_ + CCS group was significantly decreased compared to the expression in the CCl_4_ + CCS-GW4869 group, suggesting the attenuating effects of Exo-rBMMSC on hepatocyte pyroptosis (Figure 2E–I, ^#^ *p* < 0.05 vs. the CCl_4_ + CCS-GW4869 group). In addition, IL-1β and IL-18 expression levels in the CCl_4_ + CCS group were dramatically decreased (Figure 2J,K, *** *p* < 0.001 vs. the CCl_4_ group).

### 3.3. Exo-rBMMSCs Alleviated Hepatocyte Pyroptosis In Vitro

**Figure 3:**
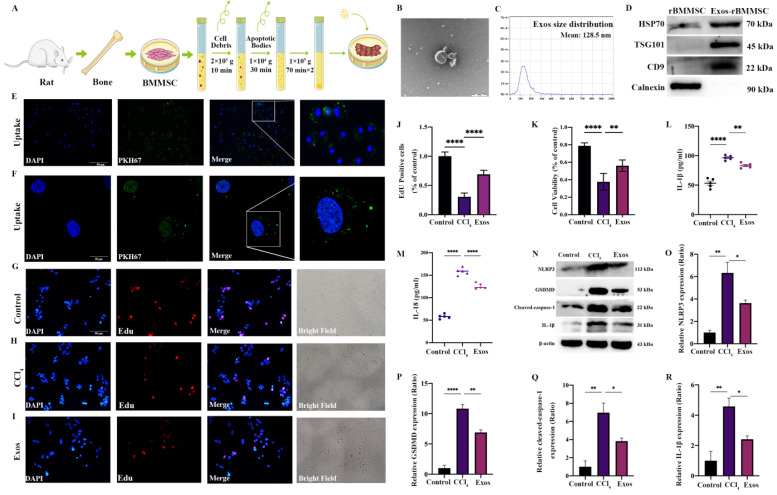
Effects of Exos-rBMMSCs on CCl_4_-induced hepatocytes. The extraction protocol of Exo-rBMMSCs is shown (**A**). TEM morphology and nanoparticle tracking analysis (NTA) results are shown (**B**,**C**). HSP70, TSG101, CD9 and Calnexin expression in the rBMMSC and Exos-rBMMSC groups is shown (**D**). PKH67-labelled Exo-rBMMSC were internalized by hepatocytes, as observed under a fluorescence microscope (**E**,**F**). Hepatocyte proliferation in the Exos group was dramatically increased ((**G**–**K**), ** *p* < 0.01 vs. the CCl_4_ group). IL-1β expression in the Exos group was significantly decreased ((**L**), ** *p* < 0.01 vs. the CCl_4_ group), and IL-18 expression in the Exos group was notably decreased compared to the CCl_4_ group ((**M**), **** *p* < 0.0001). NLRP3, GSDMD, cleaved-caspase-1 and IL-1β expression in the Exos group was significantly decreased compared to that in the CCl_4_ group ((**N**–**R**), * *p* < 0.05). The data are displayed as the means ± SD; n = 5 for each group.

Exo-rBMMSC were isolated by differential ultracentrifugation, as shown in Figure 3A. The morphology of Exo-rBMMSC was examined by transmission electron microscopy (TEM) and showed a saucer-like shape (Figure 3B). The concentration of Exo-rBMMSC was 1.0 × 10^10^ particles/mL, and 98% of the particles ranged from 30 nm to 200 nm (Figure 3C). Exosome-specific markers were highly expressed in Exo-rBMMSC (Figure 3D). Based on these results, we successfully purified Exo-rBMMSC. PKH67-labeled Exo-rBMMSC were internalized by hepatocytes, as shown in Figure 3E,F. Exo-rBMMSCs dramatically promoted CCl_4_-induced hepatocyte proliferation, as shown by EdU assays (Figure 3G–J, **** *p* < 0.0001 vs. the CCl_4_ group). Similar results were obtained from the CCK-8 assay, and the cell viability of the Exos group was significantly higher than that of the CCl_4_ group (Figure 3K, ** *p* < 0.01). Additionally, compared to that in the CCl_4_ group, IL-1β and IL-18 expression in the Exos group was significantly lower (Figure 3L,M, *** *p* < 0.001). In contrast to those in the CCl_4_ group, the expression levels of pyroptosis-related proteins in the Exos group were notably decreased (Figure 3N–R, * *p* < 0.05).

### 3.4. The Distribution of Exo-rBMMSC In Vivo

**Figure 4:**
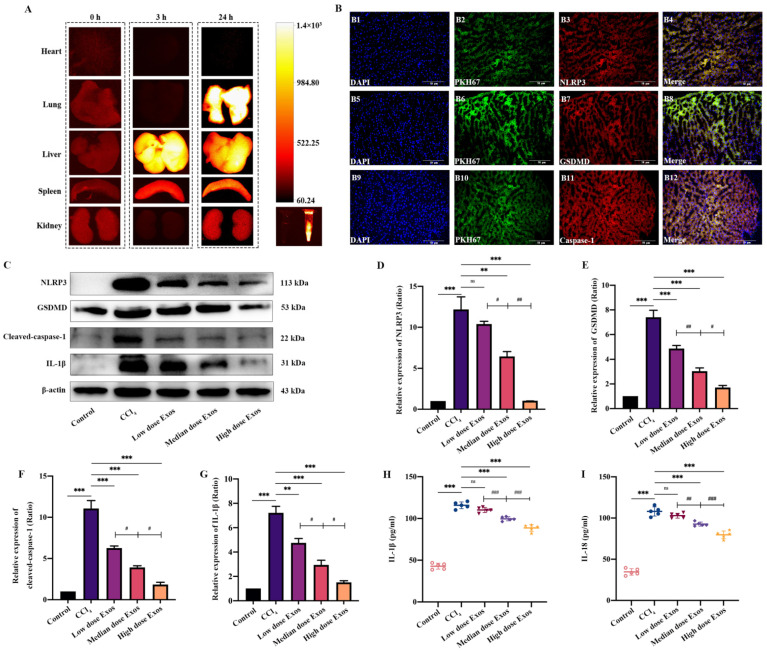
Distribution of Exos-rBMMSC and the therapeutic effect on liver cirrhosis. The distribution of Exos was examined by NIRF at 0 h, 3 h and 24 h post-injection. At 3 h post-injection, Exos were mainly concentrated in the liver and were mainly concentrated in the liver and lung at 24 h post-injection (**A**). The colocalization of NLRP3, GSDMD and caspase-1 with PKH67 dye is shown in (**B**), scale bar = 50 μm. Compared to that in the CCl_4_ group, NLRP3, GSDMD, cleaved caspase-1, and IL-1β expression in the medium and the high Exos groups was dramatically decreased ((**C**–**G**), ** *p* < 0.01). The expression of NLRP3 in the low dose Exos-rBMMSC group was not significantly different from that in the CCl_4_ group ((**D**), *p* > 0.05). The expression of GSDMD, cleaved-caspase-1 and IL-1β in the low dose Exos group was dramatically decreased compared to that in the CCl_4_ group ((**E**–**G**), ** *p* < 0.01). The expression of NLRP3, GSDMD, cleaved-caspase-1 and IL-1β in the medium dose Exos group was significantly higher than that in the high dose Exos-rBMMSC group ((**C**–**G**), ^#^ *p* < 0.05). IL-1β and IL-18 expression in the medium and high dose Exos-rBMMSC groups was dramatically lower than that in the CCl_4_ group ((**H**,**I**), *** *p* < 0.001), while the expression in the low dose Exos-rBMMSC group was not different from that in the CCl_4_ group ((**H**,**I**), *p* > 0.05). IL-1β and IL-18 expression in the medium dose Exos group was significantly lower than that in the low dose Exos group but was higher than that in the high dose Exos group ((**H**,**I**), ^##^ *p* < 0.01). The data are displayed as the means ± SD; n = 5 for each group.

DiR-labeled Exo-rBMMSC were injected through the tail vein. At 0, 3 and 24 h postinjection, the fluorescence intensities in major organs, including the heart, lung, liver, spleen and kidney, were measured by an in vivo imaging system. At 3 h postinjection, Exo-rBMMSC were mainly concentrated in the liver and spleen. At 24 h postinjection, Exo-rBMMSC migrated to the lung, liver and spleen (Figure 4A). To further confirm whether PKH67-labelled Exo-rBMMSC were taken up by injured hepatocytes, an immunofluorescence assay was performed. The colocalization of PKH67-labelled Exo-rBMMSC with NLRP3, GSDMD and caspase-1 is shown in Figure 4B.

### 3.5. Exo-rBMMSC Reduced Pyroptosis Protein Expression In Vivo

To determine the optimal dose of Exo-rBMMSC, the low dose (100 μg), the medium dose (200 μg) and the high dose (400 μg) of Exo-rBMMSC were injected via the tail vein. Four weeks postinjection, liver samples from the different groups were collected. Compared to that in the CCl_4_ group, the expression level of NLRP3 in the medium and high dose groups was significantly decreased, while that in the low dose group was not significantly different (Figure 4D, ** *p* < 0.01). NLRP3 expression in the medium dose group was significantly lower than that in low dose group and higher than that in the high dose group (Figure 4D, ^#^ *p* < 0.05). Similar results were observed for the GSDMD, caspase-1 and IL-1β loci. These proteins were expressed at significantly higher levels in the CCl_4_ group than in the other groups (Figure 4D–G, ** *p* < 0.01). The expression of these proteins in the medium dose group was dramatically lower than that in the low dose group but higher than that in the high dose group (Figure 4D–G, ^#^ *p* < 0.05). In addition, serum IL-1β and IL-18 levels showed similar results (Figure 4H,I).

### 3.6. Exo-rBMMSC Further Ameliorated Histopathological Changes

**Figure 5:**
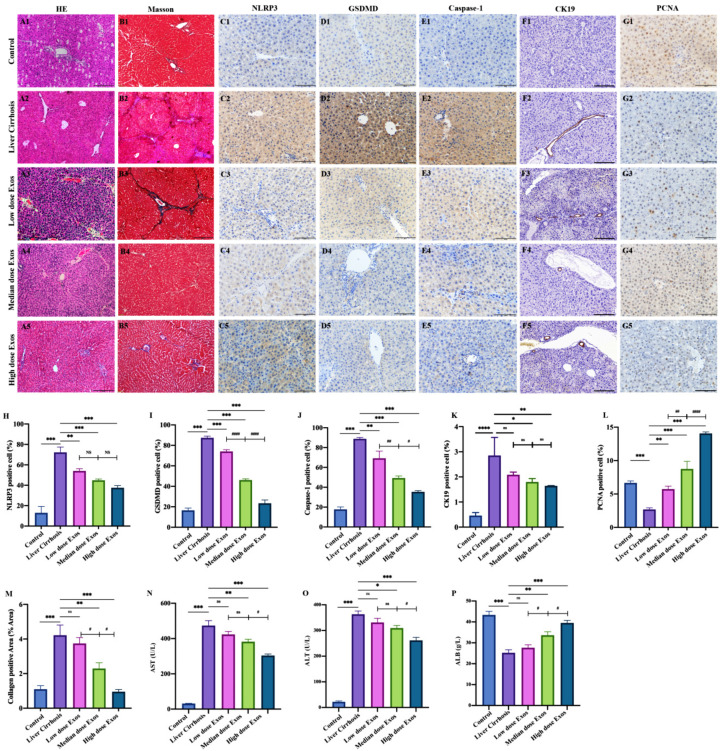
The therapeutic effect of Exo-BMMSC on liver cirrhosis was assessed in pathological sections and by measuring liver function. HE-stained sections from the control, liver cirrhosis and exosome treated groups are shown (**A**). Masson-stained sections from the different treatment groups are shown (**B**). Semi-quantitative analysis of the collagen-positive area showed that the areas in the medium dose and the high dose Exos-rBMMSC groups were significantly decreased compared with those in the liver cirrhosis group ((**M**), ** *p* < 0.01). The collagen area in the medium dose Exos group was significantly lower than that in the low dose Exos group but was higher than that in the high dose Exos group ((**M**), ^#^ *p* < 0.05). NLRP3-, GSDMD-, caspase-1-, CK19- and PCNA-positive cells were assessed by immunohistochemistry (**C**–**G**). Semi-quantitative analysis showed that NLRP3-, GSDMD-, caspase-1- and PCNA- positive cells in all the Exos treatment groups were significantly lower than those in the liver cirrhosis group ((**G**–**K**), ** *p* < 0.01). The CK19-positive cells in the low dose Exo group had no significant significance compared to the liver cirrhosis ((**K**), *p* > 0.05). The NLRP3-positive cells and the CK19- positive cells in the medium dose Exos group were not significantly different from those in the low dose and the high dose Exos groups (**H**,**K**). The numbers of GSDMD- and caspase-1-positive cells in the medium dose Exos group were dramatically lower than those in the low dose Exos group but were higher than those in the high dose Exos group ((**I**,**J**), ^#^ *p* < 0.05). The numbers of PCNA- positive cells in the medium dose Exos group were significantly higher than those in the low dose Exo group but lower than those in the high dose group ((**L**), ^##^ *p* < 0.01). AST and ALT expression in the medium dose and the high dose Exos groups was significantly decreased, but ALB expression was significantly increased compared to that in the liver cirrhosis group ((**N**–**P**), * *p* < 0.05). The expression of AST and ALT in the high dose Exos group was dramatically lower ((**N**–**O**), *** *p* < 0.001 vs. Liver cirrhosis group), while the expression of ALB was higher than that in the medium dose Exos group ((**P**), ^#^ *p* < 0.05). The data are displayed as the means ± SD.

HE-stained liver sections from the hepatic cirrhosis group showed inflammatory cell infiltration, collagen deposition and pseudolobule formation (Figure 5(A2)). Four weeks post-injection, the Exo-rBMMSC-treated groups showed pathological remission (Figure 5(A3)–(A5)). Masson-stained sections showed that 4 weeks of treatment with the medium dose and the high dose Exo-rBMMSC significantly relieved collagen deposition, while the low dose Exo-rBMMSC induced no marked improvements compared to the liver cirrhosis group (Figure 5B, ** *p* < 0.01). Similar results were observed in the semiquantitative collagen analysis; the medium and the high dose of Exo-rBMMSCs induced dramatic reductions in collagen levels compared to those in the liver cirrhosis group (Figure 5M, ** *p* < 0.01). There were no significant differences between the low dose treatment and liver cirrhosis groups (Figure 5M, *p* > 0.05).

### 3.7. Exo-rBMMSC Inhibited Hepatocyte Pyroptosis and Accelerated Proliferation

Immunohistochemical assays showed that NLRP3, GSDMD, caspase-1 and CK19 were more highly expressed in the liver cirrhosis group than in the other groups, and the levels in the medium and high dose Exo-rBMMSC groups were dramatically decreased (Figure 5C–F). Quantitative analysis showed that NLRP3-positive cells in the high dose Exo-rBMMSC group were significantly reduced compared to those in the liver cirrhosis group (Figure 5H, *** *p* < 0.001), but were not significantly different compared to those in the low and medium dose Exo-rBMMSC groups (Figure 5H, *p* > 0.05). Compared with those in the cirrhosis group, GSDMD-positive cells in the Exo-rBMMSC groups were dramatically decreased (Figure 5I, *** *p* < 0.001). GSDMD-positive cells in the high dose Exo-rBMMSC group were markedly decreased compared to those in the medium and low dose Exo-rBMMSC groups (Figure 5I, ^###^ *p* < 0.001). Similar results were observed in the number of caspase-1 positive cells (Figure 5J). CK19 positive cells in the medium dose group and the high dose group were significantly decreased compared to those in the liver cirrhosis. Comparing to the high dose group, the CK19 positive cells in the medium dose group had no significant difference (Figure 5K, *p* > 0.05). Notably, proliferating cell nuclear antigen (PCNA), which is the basic element in DNA replication and repair, was significantly increased in the medium and the high dose Exo-rBMMSC groups compared to the liver cirrhosis group (Figure 5G,L, *** *p* < 0.001).

### 3.8. Exo-rBMMSC Enhanced Liver Function

Liver function was assessed by measuring aspartate aminotransferase (AST) and alanine aminotransferase (ALT) and albumin (ALB) expression levels. Compared to those in the medium and high dose Exo-rBMMSC groups, AST and ALT expression levels in the liver cirrhosis group were markedly increased; in contrast, ALB was markedly decreased (Figure 5N–P, * *p* < 0.05). AST and ALT expression in the medium dose Exo-rBMMSC group was significantly higher than that in the high dose Exo-rBMMSC group, while there were no differences compared to that in the low dose Exo-rBMMSC group (Figure 5N,O, ^#^ *p* < 0.05). ALB expression in the medium dose Exo-rBMMSC group was markedly higher than that in the low dose Exo-rBMMSC group and dramatically lower than that in the high dose Exo-rBMMSC group (Figure 5P, ^#^ *p* < 0.05).

## 4. Discussion

Liver cirrhosis is the end stage of all types of hepatic diseases, and complications cause a substantial economic burden and affect quality of life. For treating liver cirrhosis and its complications, searching for effective therapies and exploring the pathogenesis of liver cirrhosis have been hot topics in recent years. In this study, we demonstrated that Exo-rBMMSC promoted hepatocyte proliferation and alleviated liver fibrosis by restraining hepatocyte pyroptosis.

Pyroptosis is characterized by membrane pore formation, cytolysis and the release of proinflammatory cytokines, including IL-1β, IL-18 and HMGB1 [16]. Previous studies have shown that hepatocyte pyroptosis and the release of inflammasome components induce hepatic stellate cell (HSC) activation and liver fibrosis [17,18]. Consistent with these findings, our study showed that pyroptosis-related proteins, including NLRP3, caspase-1, GSDMD, cleaved caspase-1 and IL-1β, were markedly upregulated in cirrhotic liver tissues. Additionally, we confirmed that 4 weeks of rBMMSC treatment by caudal vein injection significantly downregulated these proteins. Although the therapeutic effect of BMMSCs on liver cirrhosis has been confirmed, the clinical application of BMMSC transplantation is restrained by low differentiation efficiency, tumor formation, immunoreaction and ethical issues. To find an alternative cell-free therapy, a coculture system of rBMMSCs and hepatocytes was established, and the results indicated that the effects were mediated by a paracrine pathway.

In recent years, mounting global studies have indicated that the secretome derived from stem cells has abundant and complex biological functions [19]. Exosomes serve as central mediators of intercellular communication owing to their unique nucleic acids and proteins, which drive their utilization as cell-free therapeutics [20]. Accumulating evidence has demonstrated that exosomes derived from stem cells alleviate liver fibrosis. The transplantation of exosomes from human umbilical cord-MSCs (Exos-hucMSCs) mitigated liver fibrosis by reducing liver inflammation, collagen deposition and epithelial-to-mesenchymal transition (EMT) [21]. Exo-rBMMSCs reduced oxidative stress and accelerated liver regeneration in a liver injury model [22]. Additionally, Exo-rBMMSC showed suppressive effects by reducing liver necrotic areas and enhancing anti-inflammatory cytokines [23]. A recent study demonstrated that Exo-rBMMSC restrained liver fibrosis by inhibiting HSC activation through the Wnt/β-catenin pathway [24]. Moreover, exosome as the therapeutic cargo in liver diseases has been widely recognized, which implied a potential alternative to cell-based therapies. Exosomes produced by miR-181-5p-overexpressing adipose-derived mesenchymal stem cells (ADSCs) significantly inhibited collagen I, vimentin, α-SMA and fibronectin expression [25]. Similar research showed that circ-0000623-overexpressing ADSCs attenuated liver fibrosis by activating hepatocyte autophagy [26]. However, the optimal dose of Exo-rBMMSC for use in liver cirrhosis therapy has not been discussed. In this study, we explored the therapeutic dose of Exo-rBMMSC for liver cirrhosis. It is worth mentioning that the effect of exosomes on the rat traumatic brain injury (TBI) model was dose-dependent in a 7-day therapeutic window. Exosomes (200 μg and 100 μg per rat) significantly alleviated long-term neuroinflammation in a TBI model, while 200 μg exosomes did not provide further therapeutic effects [27]. Another study reported that exosomes from 1 × 10^8^ ADSCs had better attenuating effects on liver fibrosis than exosomes from 1 × 10^6^ and 1 × 10^7^ ADSCs [28]. According to our results, injection of both the medium dose (200 μg) and the high dose (400 μg) of Exo-rBMMSCs (per rat) via the tail vein significantly restrained hepatocyte pyroptosis and collagen deposition. Interestingly, our results showed that high dose (400 μg) Exo-rBMMSC showed a better therapeutic effect than the medium dose (200 μg) Exo-rBMMSC. The present study was the first to suggest the optimal dose of Exo-rBMMSCs for liver cirrhosis, laying an experimental foundation for clinical applications. However, there are some limitations in our study. Firstly, further studies are warranted to discover the function of molecules within Exo-rBMMSC, such as DNA, mRNA, noncoding RNA and specific growth factors. Secondly, the safe range of Exo-rBMMSC dosage was not explored in this study, which would be investigated in the future.

## 5. Conclusions

Overall, in this study, we demonstrated that Exo-rBMMSCs significantly accelerated hepatocyte proliferation and relieved liver fibrosis, and the effect may depend on restraining hepatocyte pyroptosis. More importantly, the effect of Exo-rBMMSC on hepatocytes was dose-dependent, and 400 μg of Exo-rBMMSC may be the optimal dose for treating liver cirrhosis. Our findings provide experimental data and supporting theories for clinical applications.

## Data Availability

Data are available on request from Zhang YC and Shizhu Jin.

## Abbreviations

Exos: exosomes; rBMMSCs: rat bone marrow mesenchymal stem cells; Exo-rBMMSC: exosomes derived from rat bone marrow mesenchymal stem cells; CCS: cell culture supernatant; miRNAs: microRNA; EVs-hucMSCs: EVs from human umbilical cord-MSCs; NIRF: near-infrared fluorescence; HE: hematoxylin and eosin; ADSCs: adipose-derived mesenchymal stem cells; EMT: epithelial-to-mesenchymal transition; TBI: traumatic brain injury; TEM: transmission electronic microscopy; CCK8: cytotoxicity kit-8; EdU: 5-ethynyl-2′-deoxyuridine; AST: aspartate aminotransferase; DiR: 1,1′-dioctadecyl-3,3,3′,3′-tetramethylindotricarbocyanine iodide; ALT: alanine aminotransferase; ALB: albumin; ANOVA: One-way analysis of variance.
